# The Promise of Prevention: The Effects of Four Preventable Risk Factors on National Life Expectancy and Life Expectancy Disparities by Race and County in the United States

**DOI:** 10.1371/journal.pmed.1000248

**Published:** 2010-03-23

**Authors:** Goodarz Danaei, Eric B. Rimm, Shefali Oza, Sandeep C. Kulkarni, Christopher J. L. Murray, Majid Ezzati

**Affiliations:** 1 Harvard School of Public Health, Boston, Massachusetts, United States of America; 2 Initiative for Global Health, Harvard University, Cambridge, Massachusetts, United States of America; 3 Brigham and Women's Hospital and Harvard Medical School, Boston, Massachusetts, United States of America; 4 University of California, San Francisco, California, United States of America; 5 Institute for Health Metrics and Evaluation, University of Washington, Seattle, Washington, United States of America; London School of Hygiene and Tropical Medicine, United Kingdom

## Abstract

**Background:**

There has been substantial research on psychosocial and health care determinants of health disparities in the United States (US) but less on the role of modifiable risk factors. We estimated the effects of smoking, high blood pressure, elevated blood glucose, and adiposity on national life expectancy and on disparities in life expectancy and disease-specific mortality among eight subgroups of the US population (the “Eight Americas”) defined on the basis of race and the location and socioeconomic characteristics of county of residence, in 2005.

**Methods and Findings:**

We combined data from the National Health and Nutrition Examination Survey and the Behavioral Risk Factor Surveillance System to estimate unbiased risk factor levels for the Eight Americas. We used data from the National Center for Health Statistics to estimate age–sex–disease-specific number of deaths in 2005. We used systematic reviews and meta-analyses of epidemiologic studies to obtain risk factor effect sizes for disease-specific mortality. We used epidemiologic methods for multiple risk factors to estimate the effects of current exposure to these risk factors on death rates, and life table methods to estimate effects on life expectancy. Asians had the lowest mean body mass index, fasting plasma glucose, and smoking; whites had the lowest systolic blood pressure (SBP). SBP was highest in blacks, especially in the rural South—5–7 mmHg higher than whites. The other three risk factors were highest in Western Native Americans, Southern low-income rural blacks, and/or low-income whites in Appalachia and the Mississippi Valley. Nationally, these four risk factors reduced life expectancy at birth in 2005 by an estimated 4.9 y in men and 4.1 y in women. Life expectancy effects were smallest in Asians (M, 4.1 y; F, 3.6 y) and largest in Southern rural blacks (M, 6.7 y; F, 5.7 y). Standard deviation of life expectancies in the Eight Americas would decline by 0.50 y (18%) in men and 0.45 y (21%) in women if these risks had been reduced to optimal levels. Disparities in the probabilities of dying from cardiovascular diseases and diabetes at different ages would decline by 69%–80%; the corresponding reduction for probabilities of dying from cancers would be 29%–50%. Individually, smoking and high blood pressure had the largest effect on life expectancy disparities.

**Conclusions:**

Disparities in smoking, blood pressure, blood glucose, and adiposity explain a significant proportion of disparities in mortality from cardiovascular diseases and cancers, and some of the life expectancy disparities in the US.

*Please see later in the article for the Editors' Summary*

## Abstract 2

Majid Ezzati and colleagues examine the contribution of a set of risk factors (smoking, high blood pressure, elevated blood glucose, and adiposity) to socioeconomic disparities in life expectancy in the US population.

## Editors' Summary

**Background:**

Life expectancy (a measure of longevity and premature death) and overall health have increased steadily in the United States over recent years. New drugs, new medical technologies, and better disease prevention have all helped Americans to lead longer, healthier lives. However, even now, some Americans live much longer and much healthier lives than others. Health disparities—differences in how often certain diseases occur and cause death in groups of people classified according to their ethnicity, geographical location, sex, or age—are extremely large and persistent in the US. On average, black men and women in the US live 6.3 and 4.5 years less, respectively, than their white counterparts; the gap between life expectancy in the US counties with the lowest and highest life expectancies is 18.4 years for men and 14.3 years for women. Disparities in deaths (mortality) from chronic diseases such as cardiovascular diseases (for example, heart attacks and stroke), cancers, and diabetes are known to be the main determinants of these life expectancy disparities.

**Why Was This Study Done?:**

Preventable risk factors such as smoking, high blood pressure, excessive body fat (adiposity), and high blood sugar are responsible for many thousands of deaths from chronic diseases. Exposure to these risk factors varies widely by race, state of residence, and socioeconomic status. However, the effects of these observed disparities in exposure to modifiable risk factors on US life expectancy disparities have only been examined in selected groups of people and it is not known how multiple modifiable risk factors affect US health disparities. A better knowledge about how disparities in risk factor exposure contribute to health disparities is needed to ensure that prevention programs not only improve the average health status but also reduce health disparities. In this study, the researchers estimate the effects of smoking, high blood pressure, high blood sugar, and adiposity on US life expectancy and on disparities in life expectancy and disease-specific deaths among the “Eight Americas,” population groups defined by race and by the location and socioeconomic characteristics of their county of residence.

**What Did the Researchers Do and Find?:**

The researchers extracted data on exposure to these risk factors from US national health surveys, information on deaths from different diseases in 2005 from the US National Center for Health Statistics, and estimates of how much each risk factor increases the risk of death from each disease from published studies. They then used modeling methods to estimate the effects of risk factor exposure on death rates and life expectancy. The Asian subgroup had the lowest adiposity, blood sugar, and smoking rates, they report, and the three white subgroups had the lowest blood pressure. Blood pressure was highest in the three black subgroups, whereas the other three risk factors were highest in Western Native Americans, Southern rural blacks, and whites living in Appalachia and the Mississippi Valley. The effects on life expectancy of these factors were smallest in Asians and largest in Southern rural blacks but, overall, these risk factors reduced the life expectancy for men and women born in 2005 by 4.9 and 4.1 years, respectively. Other calculations indicate that if these four risk factors were reduced to optimal levels, disparities among the subgroups in deaths from cardiovascular diseases and diabetes and from cancers would be reduced by up to 80% and 50%, respectively.

**What Do These Findings Mean?:**

These findings suggest that disparities in smoking, blood pressure, blood sugar, and adiposity among US racial and geographical subgroups explain a substantial proportion of the disparities in deaths from cardiovascular diseases, diabetes, and cancers among these subgroups. The disparities in risk factor exposure also explain some of the disparities in life expectancy. The remaining disparities in deaths and life expectancy could be the result of preventable risk factors not included in this study—one of its limitations is that it does not consider the effect of dietary fat, alcohol use, and dietary salt, which are major contributors to different diseases. Thus, suggest the researchers, reduced exposure to preventable risk factors through the implementation of relevant policies and programs should reduce life expectancy and mortality disparities in the US and yield health benefits at a national scale.

**Additional Information:**

Please access these Web sites via the online version of this summary at http://dx.doi.org/10.1371/journal.pmed.1000248.

- The US Centers for Disease Control and Prevention, the US Office of Minority Health, and the US National Center on Minority Health and Health Disparities all provide information on health disparities in the US
- MedlinePlus provides links to information on health disparities and on healthy living (in English and Spanish)
- The US Centers for Disease Control and Prevention provides information on all aspects of healthy living
- The American Heart Association and the American Cancer Society provide information on modifiable risk factors for patients and caregivers
- Healthy People 2010 is a national framework designed to improve the health of people living in the US
- The US National Health and Nutrition Examination Survey (NHANES) and the Behavioral Risk Factor Surveillance System (BRFSS) collect information on risk factor exposures in the US

## Introduction

Life expectancy disparities in the United States (US) are extremely large and have persisted over time [1]–[6]. For example, black men and women in the US live 6.3 and 4.5 years, respectively, less than their white counterparts [3]. The life expectancy gap between the counties with the highest and lowest life expectancies is about 18.4 years for men and 14.3 years for women, with even larger disparities for race–county combinations [1],[2]. Previous research has shown that disparities in mortality from chronic diseases, especially cardiovascular diseases (CVD), cancers, and diabetes, are the main determinants of life expectancy disparities by race and by county in the US, with additional effects from HIV/AIDS and homicide in men [1]–[3],[7],[8]. Disparities for diseases related to smoking and alcohol use, and those related to health care access, also seem to be determinants of mortality disparities by socioeconomic status in European countries [9].

Preventable risk factors such as smoking, elevated blood pressure, and adiposity are responsible for hundreds of thousands of chronic disease deaths in the US [10]. Data from health examination and interview surveys show that there are large differentials by race, state of residence, and socioeconomic status in exposure to these risk factors [7],[11]–[17]. To ensure that prevention policies and programs not only improve average health status but also reduce disparities, it is essential to know how much the observed disparities in risk factor exposure contribute to disparities in mortality and life expectancy. The effects of modifiable risk factors on US mortality disparities have also been analyzed in selected cohorts [18]–[20]. National analysis in the US has been limited to the effects of smoking on male mortality disparities or to the effects of multiple risk factors on disparities in self-reported health status and disease diagnosis [21]–[23]. There is currently no estimate of the effects of multiple modifiable risk factors on life expectancy disparities in the US. This is in contrast to the extensive research on the socioeconomic and health care determinants of health disparities [24]–[29]. This lack of evidence limits the ability to assess how risk factor interventions are expected to affect health disparities above and beyond their aggregate national impacts, and what combination of risk factor interventions may be used, in combination with social and health care policies, to reduce mortality disparities.

We used multiple national data sources on risk factor exposures and epidemiologic evidence on their mortality effects to quantify how much four major chronic disease risk factors affect life expectancy disparities in the US, individually and in combination. Specifically, we examined how differentials in current risk factor exposure account for disparities in life expectancy and disease-specific mortality among population subgroups based on race and the location and socioeconomic characteristics of county of residence.

## Methods

### Population Subgroups

Previous analyses have demonstrated that grouping the US population by a combination of race and county characteristics (region, median county income by race, rural versus urban status of a county, and urban homicide risk) encompass a relatively large part of the overall observed race–county life expectancy disparities, while keeping the number of subgroups tractable and their definitions constant over time [1],[2]. Based on these findings, we used eight subgroups of the US race–county combinations, referred to as the Eight Americas in a previous work [1] and defined in Table 1, as our units of analysis. The Eight Americas also provide sufficient sample size for statistically reliable estimates of risk factors and disease-specific mortality.

**Table 1:** Definitions and selected characteristics of the Eight Americas [1].

America	General Description	Population (Millions in 2005)	Per-Capita Income	Percent Completing High School	Definition
1	Asians	12.2	$21,566	80%	Asians living in counties in which Pacific Islanders make up less than 40% of total Asian population
2	Northland low-income rural whites	3.5	$17,758	83%	Whites in the northern plains and Dakotas with 1990 county-level per capita income below $11,775 (national median for whites) and population density less than 100 persons/km^2^
3	Middle America	223	$24,640	84%	All other whites not included in Americas 2 and 4, Asians not in America 1, and Native Americans not in America 5
4	Low-income whites in Appalachia and the Mississippi Valley	17	$16,390	72%	Whites in Appalachia and the Mississippi Valley with 1990 county-level per capita income below $11,775
5	Western Native Americans	1	$10,029	69%	Native American populations in the mountain and plains areas, predominantly on or near reservations
6	Black middle America	25.7	$15,412	75%	All other black populations living in counties not included in Americas 7 and 8
7	Southern low-income rural blacks	5.9	$10,463	61%	Blacks living in counties in the Mississippi Valley and the Deep South with population density below 100 persons/km^2^, 1990 county-level per capita income below $7,500 (national median for blacks), and total population size above 1,000 persons (to avoid small numbers)
8	High-risk urban blacks	7.4	$14,800	72%	Urban populations of more than 150,000 blacks living in counties with cumulative probability of homicide death between 15 and 74 years of age greater than 1.0%

Income per capita and education were calculated for race-county combinations from the 2000 US census.

### Risk Factors in the Analysis

The risk factors in this analysis were four of five leading risk factors for mortality in the US based on our recent analysis [10]: smoking, high blood pressure (measured with usual systolic blood pressure, SBP), high blood glucose (measured with usual fasting plasma glucose, FPG), and adiposity (measured with body mass index, BMI). In 2005, smoking was responsible for an estimated 467,000 deaths, high blood pressure 395,000, high BMI 216,000, and high blood glucose 190,000 [10]. Inadequate/no physical activity was the fourth leading risk factor but was not included because the available data were not of sufficient quality to directly measure or indirectly estimate exposure in the Eight Americas.

We report disparities in life expectancy and probabilities of death across the Eight Americas using both the population-weighted standard deviation (SD) of life expectancy and the difference in life expectancy between the Americas with the highest and lowest life expectancies.

### Calculating Mortality Attributable to Risk Factors

We estimated the number of deaths that would have been prevented in 2005 if past and current exposure to these risk factors had been at an alternative (lower) distribution. This can be interpreted as the excess number of deaths (or excess mortality rate) caused by the individual- and population-based determinants that have led to the observed distributions of risk factor exposure. We conducted all analyses separately by sex and age group (30–44, 45–59, 60–69, 70–79, and 80+ years), and separately for each of the Eight Americas.

Most chronic diseases are caused by multiple risk factors that act together, sometimes through overlapping pathways. For example, some CVD deaths in people who both smoke and have high SBP may be prevented by reducing either risk factor. Further, the effects of one risk factor, e.g. BMI, may be mediated partly through other risks, e.g. SBP and FPG. Therefore, simple addition of the effects of individual risk factors will generally overestimate the true combined impact of multiple factors. Our analyses systematically incorporated multicausality and mediated effects as described below.

For each America–age–sex unit of analysis, we first calculated the proportion of disease-specific deaths that would have been prevented if the exposure to these risks had been reduced to an alternative (lower) distribution; this metric is known as the population attributable fraction (PAF). The diseases included in the analysis were selected on the basis of a review of evidence on causal associations, with the sources of evidence and list of diseases with strong or convincing evidence presented in detail elsewhere [10].

In previous work, we had calculated the PAF using the distribution of individual risks in the population [10],[30] and had subsequently used a simple relationship to calculate the combined (joint) PAF for multiple risks [31]. In the current analysis, we used a computational approach that incorporated two important features of multiple risk analysis: First, the effects of BMI on CVD are mediated partly through SBP and FPG, which are also included in the analysis, with the remainder through other pathways (e.g., dyslipidemia and inflammation) [32]–[34]. Therefore, the combined effects of BMI, SBP, and FPG will be those of the latter two plus the non-SBP/FPG-mediated effect of BMI. Data sources for establishing the mediated component of the effect of BMI on CVD outcomes are described below. The second feature of multiple risks incorporated into our current analysis is their correlation, i.e., that some people have higher/lower exposure to multiple risk factors due to common socioeconomic or behavioral determinants. It is known that when risk factors are correlated, the simple approach to estimating PAFs that assumes independent distributions may be biased [35]. To incorporate risk factor correlation, we computed the PAF by summing the risks for individual BRFSS participants, weighted by their sampling probability (see Text S1 for details).

For each disease causally associated with these risk factors, the mortality rate attributable to the combined effects of risk factors was calculated by multiplying the joint PAF with the observed mortality rate in the corresponding America–age–sex unit of analysis. To calculate all-cause mortality attributable to risk factors, we summed the attributable disease-specific mortality rates in each America–age–sex unit of analysis. Mortality rates from different diseases can be added because the International Classification of Disease system assigns each death categorically to only one underlying disease cause; hence deaths from different diseases are mutually exclusive and additive.

### Calculating the Effects of Risk Factors on Life Expectancy and Probabilities of Death

We calculated life expectancy for each America using standard life table methods, and used validated demographic techniques to estimate the mortality rates in the oldest ages where both population numbers from census and the number of deaths from vital registration are less reliable [36],[37]. We calculated life tables for each sex and America using the observed age-specific mortality rates as well as the mortality rates that would be expected if risk factor exposures had been at the alternative distribution. The differences between the two sets of life expectancies measure the life expectancy gain from having reduced risk factors to the alternative distribution. Similarly, we used life table methods to calculate the effects of risk factors on the probability of dying between the ages of 15 and 60 y (denoted _45_Q_15_) and between the ages of 60 and 75 y (denoted _15_Q_60_).

### Data Sources

#### Risk factor exposure

National Health and Nutrition Examination Survey (NHANES) provides measured risk factor data by race at the national level; Behavioral Risk Factor Surveillance System (BRFSS) provides subnationally representative self-reported data on weight and height, smoking, and history of diagnosis with hypertension and diabetes. Self-reported weight and height data may be biased because of intentional misreporting. For SBP and FPG, respondents may not be aware of their risk status if they have not had recent health system encounter; after diagnosis, medication use and lifestyle modification may lower risk factor level. We used previously described and validated statistical methods [14]–[16] to combine data from NHANES and BRFSS for estimating unbiased risk factor levels in the BRFSS. BRFSS records could be assigned to the Eight Americas using race and county identifiers. We pooled the BRFSS data for 2003 and 2005 in this analysis because these years included all variables needed for predicting unbiased risk factor levels as well as county identifiers. County identifiers for 2006 and subsequent years were not available to us.

#### Disease-specific deaths

The number of deaths by underlying cause, age and sex in 2005 was obtained from the National Center for Health Statistics (NCHS), which maintains records for all deaths in the US. Although the US has automated (computerized) assignment of an International Classification of Diseases code for the underlying cause of death, the validity and comparability of cause of death statistics may be affected at the time of medical certification, especially for CVD and diabetes [38]–[40]. We adjusted for incomparability in cause of death assignment using previously described methods [38],[39]. These adjustments required information on multiple contributing causes of death and county of residence. We obtained county identifiers for all deaths in 2005 through a special request to the NCHS.

Linked mortality follow-up studies in the US have found that there is differential under- or overestimation of race-specific mortality rates [5]. This occurs because race is recorded by individuals or their families in the census and by the certifying physician or funeral facility on the death certificate. Differential recording is a potential source of bias in race-specific mortality rates and life expectancy. Studies using linked data have shown that this bias may be as much 3% among Asians, which are one of the Eight Americas. We used the National Mortality Followback Survey [5] to adjust for this bias in America 1, by age and sex. There was a corresponding reduction in deaths in America 3, the group to which Asians are most likely to be misclassified, so that the total number of deaths in the US remained constant.

#### Population

We obtained population estimates for 2005 by age, sex, race, and county of residence from the NCHS. We used post-enumeration surveys to adjust the population estimates for under-counting of Asian Americans [5]. The combination of adjustments for under-reporting of deaths and under-counting of population in census for Asian-Americans increased the mortality rate in this group (America 1) by 3%.

#### Effects of individual risk factors on disease-specific mortality

We estimated mortality effects for those diseases for which there was strong or convincing evidence of causal association, with evidence evaluated in previous work [10]. For each risk factor and disease we obtained relative risks (RRs) by age and sex from systematic reviews and meta-analyses of most recent epidemiologic studies, as described elsewhere [10].

We reviewed the evidence on whether RRs vary by race from trials and observational studies in the US and other countries (Table S1). The current evidence indicates that while the *absolute* effects (e.g., excess mortality rate) of risk factors vary by race, their *proportional* effects (i.e., RR) did not vary appreciably by race and ethnicity.

#### Effect of BMI mediated by SBP and FPG

We used a recent meta-analysis of epidemiologic studies that had estimated the effect of BMI on CVD that is mediated through SBP and FPG, and found additional studies (Table S2) that that had not been included in this meta-analysis. Based on a quantitative overview of these studies, we estimated that 50% (95% CI 30%–70%) of the excess risk (RR minus one) for the effect of BMI on CVD was mediated through SBP and FPG.

### Alternative Risk Factor Exposure Distributions

We estimated the effects of risk factors on mortality and life expectancy in the Eight Americas relative to three different alternative exposure distributions (Table 2): (1) the lowest observed exposure in any of the Eight Americas, by age (because this exposure has been achieved in at least one of the Americas, it constitutes a feasible alternative); (2) an optimal distribution in which the hazardous effects of risk factors are minimized, as described in detail elsewhere [10],[41]; and (3) a distribution whose mean equals current clinical guidelines.

**Table 2:** Risk factors in this analysis, their exposure metric, lowest observed mean in the Eight Americas, clinical guidelines, and optimal exposures.

Risk Factor^a^	Exposure Metric (Unit)	Lowest Age-Specific Mean Exposure in the Eight Americas			Clinical Guidelines^b^	Optimal Exposure Distribution c
Tobacco smoking	Current and former smoking (percent)	**Age**30–4445–5960–6970–7980+	**Current**77331	**Former**89171714	No smoking	No smoking
High blood pressure	Systolic blood pressure, SBP (mmHg)	**Age**30–4445–5960–6970–7980+	**Mean**111^d^121130134128	**SD**59131310	130 (6.78) [67]	115 (6)
Overweight-obesity (high body mass index, BMI)	BMI (kg/m^2^)	**Age**30–4445–5960–6970–7980+	**Mean**25.426.827.427.225.4	**SD**4.14.22.52.813.7	25 (1.2)	21 (1)
High blood glucose	Fasting plasma glucose, FPG (mg/dl)	**Age**30–4445–5960–6970–7980+	**Mean**899710510297	**SD**713191715	100 (5.9) [68]	88 (5.2)

a See a previous national analysis of risk factor effects for a list of disease outcomes causally associated with each risk factor [10].

b The means (SD) of the normal distribution are reported. We used the threshold used in the recent clinical guidelines as the mean. The SD was estimated using the coefficient of variation for the optimal exposure distribution of the same risk factor.

c The epidemiologic evidence for selection of optimal distribution is described in a previous work [10]. The primary criterion was the level to which randomized trials and observational studies indicate benefits of lowering exposure continue. The mean (SD) of the normal distribution are reported here.

d The lowest observed mean SBP in this age group was lower than the mean of the optimal distribution, i.e., the level to which benefits of lowering exposure have been observed in current epidemiologic studies. We did not assign any benefits to exposures lower than the optimal distribution.

### Analyses of Uncertainty

We quantified the uncertainty due to sampling variability using a simulation approach. In each of 1,000 simulation rounds, for each America–age–sex analysis unit, we drew (1) a random sample of the participants in the BRFSS; this sample was drawn with replacement and the sample size was equal to the original number of participants with no missing risk factor data (bootstrapping) [42]); (2) a RR for each disease causally associated with risk factors from a log-normal distribution whose standard error was from epidemiologic studies [10]; (3) the proportion of the excess risk of BMI mediated through SBP and FPG from a normal distribution with mean of 0.5 and SD of 0.1; and (4) a disease-specific mortality rate for each disease causally associated with risk factors, with the distribution of mortality rate obtained as described elsewhere [2]. The 1,000 simulated draws were used to estimate the sampling uncertainty for mortality and life expectancy impacts of risk factors.

All analyses were conducted using STATA 10.1 (StataCorp, Texas, USA). The simulations were run on the Orchestra Research Computing Custer supported by the Harvard Medical School Research Information Technology Group.

## Results

### Disparities in Risk Factor Exposure

There were substantial disparities in exposure to these four risk factors across the Eight Americas (Table 3). Asian American men and women (America 1) had the lowest BMI, FPG, and smoking, while whites had the lowest mean SBP. The highest SBP was observed in blacks, especially those in the rural South (America 7), whose mean SBP was 5–7 mmHg higher than whites in different age and sex groups. The highest mean BMI was in western Native American men (age-standardized mean 30.0 versus 26.7 kg/m^2^ in Asians) and Southern low-income rural black women (age-standardized mean 33.9 versus 26.2 kg/m^2^ in Asians). Mean FPG across the Eight Americas generally followed the same pattern as BMI. Western Native Americans (America 5) and low-income whites in the Appalachia and Mississippi Valley (America 4) had the highest smoking prevalence, with about 30% of men and women currently smoking. Smoking prevalence was also relatively high among blacks.

**Table 3:** Exposure to risk factors by sex and age group in the Eight Americas.

Age–Sex Group	America ^a^	SBP, mmHg	BMI, kg/m^2^	FPG, mg/dl	Current Smoking, %	Former Smoking, %
**Male 30–59 y**	Asians (America 1)	125 (1.7)	**26.6 (0.39)**	**99 (0.8)**	**15 (3.5)**	23 (4.3)
	Northland low-income rural whites (America 2)	**122 (1.0)**	28.7 (0.28)	100 (0.4)	24 (2.7)	26 (2.6)
	Middle America (America 3)	123 (0.2)	28.6 (0.08)	101 (0.2)	24 (0.6)	**27 (0.6)**
	Low-income whites in Appalachia and Mississippi Valley (America 4)	**122 (0.6)**	29.1 (0.19)	102 (0.3)	**35 (1.8)**	26 (1.5)
	Western Native Americans (America 5)	127 (2.7)	**30.2 (0.78)**	**104 (1.2)**	33 (6.6)	24 (5.8)
	Black middle America (America 6)	128 (1.0)	29.3 (0.32)	102 (0.6)	28 (2.6)	**18 (2.2)**
	Southern low-income rural blacks (America 7)	**129 (1.3)**	29.7 (0.44)	**104 (1.0)**	34 (3.7)	19 (2.7)
	High-risk urban blacks (America 8)	**129 (1.5)**	29 (0.59)	101 (0.8)	31 (4.5)	19 (4.0)
**Male ≥60 y**	Asians (America 1)	135 (4.4)	**27 (0.81)**	**106 (1.9)**	**5 (3.5)**	**35 (11.7)**
	Northland low-income rural whites (America 2)	**133 (1.2)**	28.6 (0.34)	110 (1.0)	11 (2.3)	**59 (3.7)**
	Middle America (America 3)	**133 (0.3)**	27.9 (0.09)	109 (0.3)	11 (0.6)	56 (1.0)
	Low-income whites in Appalachia and Mississippi Valley (America 4)	**133 (0.8)**	27.9 (0.21)	110 (0.6)	14 (1.4)	56 (2.2)
	Western Native Americans (America 5)	138 (4.0)	**29.4 (1.14)**	**116 (3.6)**	**21 (9.2)**	40 (9.8)
	Black middle America (America 6)	138 (2.0)	28.3 (0.52)	112 (1.4)	19 (4.3)	45 (6.1)
	Southern low-income rural blacks (America 7)	**140 (2.0)**	28.7 (0.57)	113 (1.8)	17 (3.7)	44 (5.6)
	High-risk urban blacks (America 8)	138 (2.9)	28 (0.78)	110 (2.1)	**21 (5.9)**	39 (7.5)
**Female 30–59 y**	Asians (America 1)	119 (1.6)	**25.6 (0.48)**	**93 (0.7)**	**7 (2.6)**	**7 (2.2)**
	Northland rural low-income whites (America 2)	**117 (1.0)**	29.2 (0.44)	95 (0.3)	23 (2.3)	22 (2.3)
	Middle America (America 3)	**117 (0.2)**	28.6 (0.1)	96 (0.1)	22 (0.5)	**23 (0.5)**
	Low-income whites in Appalachia and Mississippi Valley (America 4)	**117 (0.5)**	30.3 (0.26)	98 (0.3)	32 (1.4)	18 (1.1)
	Western Native Americans (America 5)	122 (2.0)	31.1 (0.92)	**101 (1.3)**	**36 (6.2)**	14 (3.4)
	Black middle America (America 6)	123 (0.8)	32.7 (0.42)	98 (0.5)	22 (1.8)	15 (1.5)
	Southern low-income rural blacks (America 7)	**124 (1.1)**	**34.3 (0.52)**	100 (0.7)	19 (2.1)	12 (1.6)
	High-risk urban blacks (America 8)	122 (1.3)	32.3 (0.68)	97 (0.6)	24 (3.0)	15 (2.4)
**Female ≥60 y**	Asians (America 1)	143 (4.8)	**27.6 (1.37)**	**103 (2.4)**	**3 (2.0)**	21 (7.2)
	Northland low-income rural whites (America 2)	**139 (1.2)**	29.7 (0.39)	104 (0.6)	8 (1.5)	27 (2.7)
	Middle America (America 3)	**139 (0.3)**	28.9 (0.12)	104 (0.2)	11 (0.5)	34 (0.7)
	Low-income whites in Appalachia and Mississippi Valley (America 4)	**139 (0.6)**	29.2 (0.25)	105 (0.4)	14 (1.1)	26 (1.4)
	Western Native Americans (America 5)	140 (3.6)	30.1 (1.79)	108 (2.9)	**15 (5.7)**	**36 (10.1)**
	Black middle America (America 6)	143 (1.6)	31.9 (0.67)	108 (1.1)	14 (2.7)	27 (3.4)
	Southern low-income rural blacks (America 7)	**144 (1.5)**	**32.7 (0.7)**	**110 (1.3)**	10 (2.4)	**20 (3.0)**
	High-risk urban blacks (America 8)	**144 (2.3)**	31.0 (0.87)	106 (1.6)	12 (3.2)	32 (5.5)

Table shows mean (standard error) for SBP, BMI, FPG, and prevalence (standard error) for smoking. The lowest and highest mean exposures in each age-sex group are shown in bold font. Within each age group, exposures are age-standardized to the 2005 US population.

### Effects of Risk Factors on Life Expectancy in the Eight Americas

**Figure 1:**
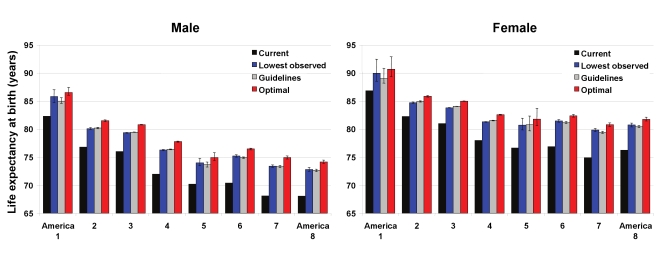
Current life expectancy at birth and life expectancy under three alternative risk factor distributions in the Eight Americas for men and women. See definitions of the Eight Americas in Table 1. The means and 95% confidence intervals for life expectancy under the three alternative distributions was estimated using simulations as described in Methods. Because the number of simulations was finite, the means may be slightly different from the numbers reported in Table 4 and the text. This difference is in the order of 0.1% and does not affect our conclusions.

In 2005, national life expectancy in the US was 75.1 y for men and 80.3 for women. Asians had the highest life expectancy at birth in the Eight Americas, reaching 86.8 y for women and 82.3 for men (Figure 1); these are, respectively, about 1 and 2 y higher than the highest life expectancies in the world (Japan for women and San Marino for men). Blacks had the lowest life expectancy in both men (68.1 y in the rural South and high-risk urban areas) and women (74.9 y in the rural South). There was a declining life expectancy gradient from America 1 to Americas 7 and 8, with the 14- and 12-y gaps in men and women equal to those observed between middle- and high-income countries worldwide [1]. The population-weighted SD of the Eight Americas life expectancies was 2.7 y for men and 2.1 y for women.

Nationally, the four preventable risk factors have lowered life expectancy by an estimated 4.9 y for men and by 4.1 y for women. Had these four risk factors been reduced to their optimal distributions in all groups, gains in life expectancy would have been larger in the Americas with currently low life expectancy, although there would be benefits in other groups also (Figure 1). For example, Southern low-income rural black men and women (America 7) would have gained 6.7 and 5.7 y, respectively, versus 4.1 and 3.6 y in Asians (America 1). The life expectancy benefits were larger for men than for women in all Americas, except in western Native Americans (4.6 y for men versus 5.0 for women). Among whites, the largest gains were in Appalachia and Mississippi Valley (America 4), where men and women would gain 5.7 and 4.5 y life expectancy if risk factor exposures were at optimal levels. Had risk factor levels been at their optimal distributions in all Americas, the population weighted SD of life expectancies would be lowered by 0.50 y (18%) in men and 0.45 y (21%) in women; life expectancy gaps between the best- and worst-off Americas would be lowered by 1.9 y (13%; America 1 versus America 8) in men and 2.0 y (17%; America 1 versus America 7) in women.

The life expectancy gains under the other two more realistic alternative exposure distributions were smaller than those under optimal distributions, by 0.7–1.5 y in different Americas when the lowest observed exposure by age was used and by 1.0–1.6 y when the guidelines were met (Figure 1). These more modest risk factor reductions would nonetheless have larger benefits for those Americas with currently lower life expectancy, and hence reduce life expectancy disparities. These alternative risk factor distributions would lower population-weighted SD by 0.44–0.47 y, about the same as those expected when risks were lowered to their optimal levels.

When risk factors were analyzed individually, removing smoking would have led to the largest gains in life expectancy in men in all Eight Americas, and in white and Native American women (Table 4). Smoking reduction alone accounted for 42%–58% of the years gained by all four risks in men and 12%–46% in women in these Americas (noting that the effects of individual risk factors on life expectancy are not additive due to multicausality and competing risk from other diseases). Lowering blood pressure to its optimal distribution would have achieved between 27% (men in America 4) and 69% (women in America 1) of the benefits of all four risk factors. The largest benefit from any single risk factor among black women was from lower blood pressure, alone explaining about one-half of the life expectancy gain from all four risks (47%–49%). Adiposity was the second single most important risk factor in black women (40%–48%).

**Table 4:** Life expectancy gains (in years) expected by reducing individual risk factors to their optimal distributions.

America ^a^	Sex	Overall Gain, y	SBP	BMI	FPG	Smoking
**National**	Male	4.9	1.5 (31)	1.3 (26)	0.5 (10)	2.5 (52)
	Female	4.1	1.6 (39)	1.3 (31)	0.3 (7)	1.8 (43)
**Asians (America 1)**	Male	4.1	1.5 (38)	1.0 (26)	0.4 (10)	1.7 (42)
	Female	3.6	2.5 (69)	0.5 (15)	0.1 (4)	0.4 (12)
**Northland low-income rural whites (America 2)**	Male	4.7	1.4 (29)	1.2 (26)	0.5 (10)	2.4 (51)
	Female	3.6	1.4 (39)	1.2 (32)	0.3 (7)	1.4 (39)
**Middle America (America 3)**	Male	4.7	1.4 (30)	1.2 (26)	0.5 (10)	2.4 (52)
	Female	4.0	1.4 (37)	1.1 (29)	0.3 (7)	1.8 (45)
**Low-income whites in Appalachia and Mississippi (America 4)**	Male	5.7	1.5 (27)	1.5 (26)	0.6 (11)	3.3 (58)
	Female	4.5	1.6 (35)	1.4 (32)	0.4 (8)	2.1 (46)
**Western Native Americans (America 5)**	Male	4.6	1.5 (32)	1.6 (34)	0.5 (11)	2.4 (51)
	Female	5.0	1.6 (33)	1.8 (36)	0.4 (9)	2.1 (42)
**Black middle America (America 6)**	Male	6.0	2.3 (38)	1.8 (30)	0.6 (11)	2.7 (45)
	Female	5.3	2.5 (48)	2.3 (43)	0.5 (10)	1.6 (30)
**Southern low-income rural blacks (America 7)**	Male	6.7	2.6 (40)	2.1 (32)	0.8 (12)	3.1 (46)
	Female	5.7	2.8 (49)	2.7 (48)	0.7 (11)	1.5 (26)
**High-risk urban blacks (America 8)**	Male	6.0	2.6 (43)	1.9 (32)	0.6 (10)	2.6 (43)
	Female	5.4	2.5 (47)	2.2 (40)	0.4 (8)	1.8 (34)

Numbers in brackets show the gain as % of gain expected if all risk four risk factors are reduced; percents add to more than 100% because of multi-causality and because life expectancy is affected by competing risk from other diseases.

### Effects of Risk Factors on Disease-Specific Probabilities of Death

**Figure 2:**
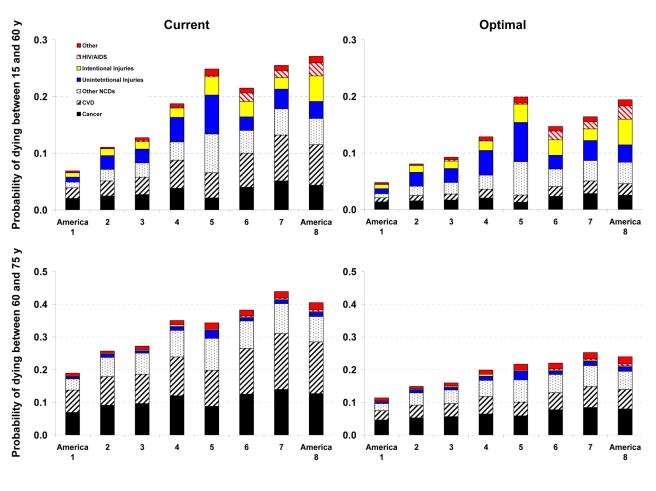
Probability of death from different medical causes between 15 and 60 years of age (_45_Q_15_) and between 60 and 75 years of age (_15_Q_60_) in the current and optimal distributions of risk factors in the Eight Americas in men. See definitions of the Eight Americas in Table 1.

**Figure 3:**
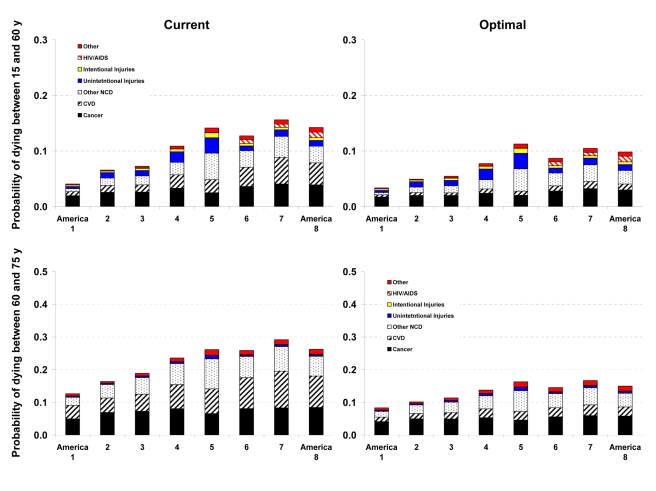
Probability of death from different medical causes between 15 and 60 years of age (_45_Q_15_) and between 60 and 75 years of age (_15_Q_60_) in the current and optimal distributions of risk factors in the Eight Americas in women. See definitions of the Eight Americas in Table 1.

Western Native Americans (America 5) and low-income blacks in the rural South (America 7) and high-risk urban areas (America 8) had the highest probability of dying between the ages of 15 to 60 y (_45_Q_15_) in the year 2005 (Figures 2 and 3). In these three Americas, about one out of four men and one out of six women who survived to age 15 are expected to die before their 60th birthday (versus 4% for men and 7% for women in America 1). Most of these deaths were caused by CVD and cancers, except in Native Americans, among whom injuries, diabetes, liver cirrhosis, digestive diseases, and alcohol use disorders also contributed substantially to young and middle-aged deaths. HIV/AIDS continued to be an important cause of death in young and middle-aged blacks despite the availability of efficacious treatments. The survival advantage of Asians and whites (except those in America 4) continued into older ages, with Asians who survive to their 60th birthday having a 19% (men) and 13% (women) probability of dying before their 75th birthday (_15_Q_60_), versus 44% and 29% for Southern rural blacks (Figures 2 and 3).

Reducing smoking, SBP, FPG, and BMI to their optimal distributions would have improved survival in all Eight Americas, with the largest benefits in those that currently have the highest probabilities of death. For example, the disparity in male _45_Q_15_ between the highest and the lowest probability of death in 2005 would decline from 0.20 to 0.15; similarly, the disparity in female _45_Q_15_ between highest and lowest probability of death would decline from 0.12 to 0.08 if these risks were at their optimal distributions. There would also be a reduction in survival disparities in older adults (_15_Q_60_).

The largest disparity effects of these risk factors were on CVD and diabetes mortality. CVD and diabetes mortality disparities in different age and sex groups in the optimal risk factor scenario would be 69%–80% smaller than their current levels. Similarly, cancer mortality disparities would be 29%–50% lower. Among cancers, breast, colorectal, and residual lung cancer deaths were the main source of remaining disparities, with their rates largest among blacks and in low-income whites in Appalachia and Mississippi Valley (America 4).

## Discussion

Life expectancy disparities in the US, measured in relation to individual and community characteristics, are enormous by international standards. The Eight Americas encompass a large part of US life expectancy disparities, while forming easily identifiable subgroups of the US population. We have shown that a small number of preventable risk factors such as smoking, high blood pressure, elevated blood glucose, and adiposity are the leading risk factors for mortality in the US [10]. The results from the current analysis suggest that these risk factors also contribute to the mortality disparities across the Eight Americas, especially for CVD and cancers. Therefore, had these risk factors been reduced to their optimal levels or even to the commonly used guidelines, there would be both aggregate health benefits and a considerable decline in life expectancy disparities. Our conclusions on the role of these risk factors on life expectancy disparities across the Eight Americas were not sensitive to the specific disparity metric used (for a discussion of disparity metrics see [43]).

Analyses of disease-specific probabilities of death identified injuries, HIV/AIDS (especially for men), and selected noncommunicable diseases as those that accounted for disparities that remained after risk factors were reduced to optimal levels. Once we removed deaths from homicide, road traffic injuries, and HIV/AIDS in addition to the effects of risk factors, life expectancy further improved with larger benefits to the Americas that currently have lower life expectancy, especially for men. For example, reducing risk factors to their optimal levels and removing these three medical causes of death increased male life expectancy in Americas 5, 7, and 8 by 7.3–9.4 y (compared with 4.6–6.7 y when only risk factors are reduced). Yet even CVD mortality had a residual gradient, albeit substantially smaller, after four of its most salient risk factors were removed, with a clear survival advantage among Asians (Figures 2 and 3). The reasons for this residual advantage may be risk factors not included in our analysis (e.g., lower lipids as a result of dietary composition or use of statins, psychosocial factors, etc.) or disparities in health care access and quality of care. These factors could not be estimated in the BRFSS, but should be the subject of future data collection and research. Further, the benefits of reducing current exposure occurs over time and requires additional analysis of time-dependent effects. Most of the benefits nonetheless seem to occur within about 5 y for cardiovascular risk factors [44]; even for the effects of smoking on cancers and chronic respiratory diseases, which have longer periods of risk reversibility, 75% or more of the benefits of cessation occur by about 15 y [45].

Our results on the effect of multiple preventable risk factors on life expectancy disparities at the national level are supported by those from analyses in specific cohorts, which were not nationally representative. For example, in the Atherosclerosis Risk in Communities (ARIC) Study, blacks and whites had nearly identical CVD incidence rates after adjustment for smoking, blood pressure, cholesterol and glucose [18]. Analyses of the Multiple Risk Factor Intervention Trial found that adjustment for major CVD risk factors reduced the differences in CVD mortality although there was a statistically significant remaining difference [19],[20]. A recent reanalysis of the Whitehall follow-up study found that interventions for the same risk factors as the ARIC study were expected to reduce coronary heart disease mortality differentials between occupational classes by 86% [46]. Finally, the Korean National Health and Nutrition Examination Survey follow-up study also found that absolute socioeconomic mortality inequalities could be substantially reduced if either behavioral (smoking, alcohol use, and physical inactivity) or metabolic risks (blood pressure, fasting serum glucose, and serum total cholesterol) were improved [47],[48]. Some of these studies found larger effects of risk factors on disparities that those in our analysis, possibly due to the inclusion of other risks (e.g., lipids, physical inactivity) and indicators such as income and education that either directly or through other metabolic, dietary, and lifestyle factors affect mortality disparities. National analysis in the US estimated that 58% of disparities in total mortality among young and middle-age men was due to smoking; there also seems to be some effect of risk factors on disparities in self-reported disease diagnoses and health status (noting that self-reported health status is commonly measured with bias, error, and incomparability) [21]–[23],[49].

Beyond its innovation in assessing the effects of modifiable risk factors on the national US life expectancy disparities, our analysis has several strengths. Our PAF calculations incorporated multicausality and mediated effects, with parameters from systematic or comprehensive reviews of epidemiologic studies. We calculated PAFs for multiple risk factors using individual level exposure data, thereby eliminating the need to parameterize the joint distribution of risk factors and make strong assumptions about the shape of the distributions and their correlations. Our outcome variables were life expectancy and probabilities of death, which incorporate competing risks from other diseases using life table methods. Further, life tables were estimated separately for the Eight Americas and by sex because they have distinct patterns of competing risks. Finally, we quantified the uncertainty as a result of the sampling variability in exposure, RRs, mediated effects of BMI, and disease-specific mortality rates.

Population-level analyses like ours also have limitations. First, the BRFSS does not provide data or indicators of sufficient detail and quality on alcohol use, blood lipids, relevant dietary risk factors (e.g., salt and various fats), and physical activity in the Eight Americas. Therefore, these risk factors could not be included in our analysis, even though they may have significant variation by race and/or region [12],[50]–[52]. Using NHANES 2003–2006 data at the national level, the combined effects of LDL cholesterol and the four risk factors in our analysis on life expectancy would be 0.1 y higher for men and women than that of these four risk factors alone. The difference between the effects of the two clusters of risk factors is small despite the fact that LDL cholesterol is an important risk factor for CVD mortality; rather, because of multicausality, the combined PAF for the effects of multiple risk factors grows by progressively smaller amounts with each additional risk factor, even when its individual effect is relatively large. Further, mean age-standardized LDL cholesterol among blacks was only 1 mg/dl (<1%) higher than whites for men; black women had lower cholesterol than whites by 7 mg/dl (∼6%), indicating that its contribution to disparities may be modest across groups other than Asians. Harmful alcohol use is an important risk factor for injuries and diseases such as cirrhosis, which had substantial disparity in the Eight Americas [53].

The second limitation of our analysis arises from the fact that the possibility of effect size modification by race cannot be ruled out, even though the current evidence is generally consistent with RRs being similar by race. RR differences may be especially relevant for smoking, for which factors such as smoking intensity and duration of smoking or smoking cessation may influence RRs. Third, because BRFSS does not measure SBP, FPG, weight, and height, we applied validated statistical models to NHANES and BRFSS data to predict these variables and correct for bias in self-reported data [14]–[16]. While this is an innovative use of multiple data sources for subnational risk factor measurement, with relatively high predictive power, it could use only those predictors that were measured in NHANES and BRFSS using consistent or comparable questionnaires. There was unexplained variation in the model that could result in underestimating exposure variability across the Eight Americas [15]. Hence our results should be considered conservative estimates of the effects of risk factors on mortality disparities. Further, these prediction models result in additional uncertainty beyond sampling error, making the reported uncertainty intervals in Figure 1 an underestimate of the true uncertainty. Fourth, the combined effects of the four risk factors included in our analysis may follow a model different fromthat presented in Text S1. For example, a part of the effect of smoking on cardiovascular diseases may be mediated through blood pressure and/or glucose. A sensitivity analysis showed that 25% of the effects of smoking on cardiovascular outcomes were mediated through these factors, our estimated effects on lifeexpectancy levels and disparities would change by less than 0.06 y.

A key feature of our analysis is using the Eight Americas, which are based on race, and on county location and socioeconomic characteristics. As discussed in previous work [1],[2], using county and race–county combinations as units of analysis has allowed us to examine mortality disparities in consistent, comparable, and easily identifiable units over decades, but does not capture within-county variations in risk factor exposure and mortality. Finally, we could not include individuals with Hispanic ethnicity as a separate America. Previous analyses have shown that Hispanic ethnicity is significantly under-recorded on death certificates, leading to implausibly high life expectancies when combined with population estimates from census using self-reported Hispanic ethnicity [54],[55]. Future analyses should attempt to adjust for this mortality undercount, or conduct analyses for Hispanics in regions where mortality undercount is likely to be small, e.g., in states with large Hispanic population [56].

Our results demonstrate that a small number of risk factors for chronic diseases account for a noticeable part of the disparities in life expectancy in the US, with the largest contributions from smoking and high blood pressure. These disparity effects influence young and middle-aged adults, as well as older adults, with the largest effects on CVD, diabetes, and some cancers. The report of the WHO Commission on Social Determinants of Health has called attention to distribution of money, power, and resources as the underlying sources of health disparities, but has also emphasized the need to focus on common risk factors for chronic diseases with known and effective interventions [57]. Similarly the most recent House of Commons Health Committee Report in the UK identified three groups of causes for health inequalities: access to health care, socioeconomic factors, and lifestyle factors [58]. The essential question is therefore how to use disease prevention to improve health and reduce health disparities together with policies that aim to reduce socioeconomic disparities, reform health care, and improve quality of care.

An essential first step in achieving the aggregate and disparity promises of prevention is to discard a dominant view in the US that behavioral, dietary, and metabolic risk factors are either personal choices and responsibilities or are in the domain of clinical practice and hence only a subject of health education and physician advice for individuals. Rather, we must identify, implement, and rigorously evaluate effective population-based and personal interventions that can reduce these preventable risk factors or mitigate their effects on disease outcomes (see, for example, the reviews commissioned by the Robert Wood Johnson Foundation on disparities in CVD and diabetes [59],[60]). Few or no current interventions have been effective in reducing overweight and obesity at the population level, emphasizing the need to develop and test new creative and ambitious interventions. Diabetes prevention through lifestyle and pharmacological interventions has been efficacious in trials [61] but should be evaluated in community settings. Smoking and blood pressure both have efficacious and cost-effective interventions, and have successfully been lowered in the adult US population as a whole for decades. These interventions need to reach population subgroups and counties where smoking and blood pressure remain high. Salt intake is an important predictor of population blood pressure [62],[63], and regulating and reducing salt in prepared and packaged food is an effective population-level intervention [64]; screening and use of antihypertensives or combination therapy to reduce blood pressure and cardiovascular risk is also cost-effective [65] and should be scaled up as a part of expanding and improving primary care in the context of US health reform. A recent systematic review of smoking interventions hypothesized that population-level interventions have the potential to reduce disparities in smoking [66]. Yet in practice, US risk factor trends have at best had a mixed performance in terms of reducing exposure disparities [17]. Therefore, both national versus local and aggregate versus disparity effects should determine the design, implementation, and evaluation of policies and programs that aim to reduce risk factor exposure.

## Supporting Information

Table S1 Evidence on effect size modification by race.
(0.07 MB DOC)

Table S2 Studies on the proportion of BMI-CVD excess risk mediated through blood pressure and blood glucose/diabetes.
(0.06 MB DOC)

Text S1 Calculating PAF for multiple risks, including mediated effects.
(0.04 MB DOC)

## Abbreviations

ARIC: Atherosclerosis Risk in Communities
BMI: body mass index
BRFSS: Behavioral Risk Factor Surveillance System
CVD: cardiovascular diseases
FPG: fasting plasma glucose
NCHS: National Center for Health Statistics
NHANES: National Health and Nutrition Examination Survey
PAF: population attributable fraction
RR: relative risk
SBP: systolic blood pressure

## References

[1] Murray CJ, Kulkarni SC, Michaud C, Tomijima N, Bulzacchelli MT (2006). Eight Americas: investigating mortality disparities across races, counties, and race-counties in the United States.. PLoS Med.

[2] Ezzati M, Friedman AB, Kulkarni SC, Murray CJ (2008). The reversal of fortunes: trends in county mortality and cross-county mortality disparities in the United States.. PLoS Med.

[3] Harper S, Lynch J, Burris S, Davey SG (2007). Trends in the black-white life expectancy gap in the United States, 1983-2003.. JAMA.

[4] Singh GK, Siahpush M (2006). Widening socioeconomic inequalities in US life expectancy, 1980-2000.. Int J Epidemiol.

[5] Hahn RA, Eberhardt S (1995). Life expectancy in four U.S. racial/ethnic populations: 1990.. Epidemiology.

[6] Krieger N, Rehkopf DH, Chen JT, Waterman PD, Marcelli E (2008). The fall and rise of US inequities in premature mortality: 1960-2002.. PLoS Med.

[7] Cooper R, Cutler J, svigne-Nickens P, Fortmann SP, Friedman L (2000). Trends and disparities in coronary heart disease, stroke, and other cardiovascular diseases in the United States: findings of the national conference on cardiovascular disease prevention.. Circulation.

[8] Wong MD, Shapiro MF, Boscardin WJ, Ettner SL (2002). Contribution of major diseases to disparities in mortality.. N Engl J Med.

[9] Mackenbach JP, Stirbu I, Roskam AJ, Schaap MM, Menvielle G (2008). Socioeconomic inequalities in health in 22 European countries.. N Engl J Med.

[10] Danaei G, Ding EL, Mozaffarian D, Taylor B, Rehm J (2009). The preventable causes of death in the United States: comparative risk assessment of dietary, lifestyle, and metabolic risk factors.. PLoS Med.

[11] Cowie CC, Rust KF, Byrd-Holt DD, Eberhardt MS, Flegal KM (2006). Prevalence of diabetes and impaired fasting glucose in adults in the U.S. population: National Health And Nutrition Examination Survey 1999-2002.. Diabetes Care.

[12] Ford ES, Li C, Pearson WS, Zhao G, Mokdad AH (2008). Trends in hypercholesterolemia, treatment and control among United States adults.. Int J Cardiol. E-pub ahead of print.

[13] Lloyd-Jones D, Adams R, Carnethon M, De SG, Ferguson TB (2009). Heart disease and stroke statistics–2009 update: a report from the American Heart Association Statistics Committee and Stroke Statistics Subcommittee.. Circulation.

[14] Ezzati M, Martin H, Skjold S, Vander HS, Murray CJ (2006). Trends in national and state-level obesity in the USA after correction for self-report bias: analysis of health surveys.. J R Soc Med.

[15] Ezzati M, Oza S, Danaei G, Murray CJ (2008). Trends and cardiovascular mortality effects of state-level blood pressure and uncontrolled hypertension in the United States.. Circulation.

[16] Danaei G, Friedman AB, Oza S, Murray CJ, Ezzati M (2009). Diabetes prevalence and diagnosis in US states: analysis of health surveys.. Popul Health Metr.

[17] Kanjilal S, Gregg EW, Cheng YJ, Zhang P, Nelson DE (2006). Socioeconomic status and trends in disparities in 4 major risk factors for cardiovascular disease among US adults, 1971-2002.. Arch Intern Med.

[18] Hozawa A, Folsom AR, Sharrett AR, Chambless LE (2007). Absolute and attributable risks of cardiovascular disease incidence in relation to optimal and borderline risk factors: comparison of African American with white subjects–Atherosclerosis Risk in Communities Study.. Arch Intern Med.

[19] Thomas AJ, Eberly LE, Davey SG, Neaton JD, Stamler J (2005). Race/ethnicity, income, major risk factors, and cardiovascular disease mortality.. Am J Public Health.

[20] Davey SG, Neaton JD, Wentworth D, Stamler R, Stamler J (1998). Mortality differences between black and white men in the USA: contribution of income and other risk factors among men screened for the MRFIT. MRFIT Research Group. Multiple Risk Factor Intervention Trial.. Lancet.

[21] Jha P, Peto R, Zatonski W, Boreham J, Jarvis MJ (2006). Social inequalities in male mortality, and in male mortality from smoking: indirect estimation from national death rates in England and Wales, Poland, and North America.. Lancet.

[22] Lantz PM, Lynch JW, House JS, Lepkowski JM, Mero RP (2001). Socioeconomic disparities in health change in a longitudinal study of US adults: the role of health-risk behaviors.. Soc Sci Med.

[23] Avendano M, Glymour MM, Banks J, Mackenbach JP (2009). Health disadvantage in US adults aged 50 to 74 years: a comparison of the health of rich and poor Americans with that of Europeans.. Am J Public Health.

[24] Adler N, Marmot M, McEwan B, Stewart J (1999). Socioeconomic status and health in industrial nations: social, psychological, and biological pathways..

[25] Committee on the Consequences of Uninsurance, Board on Health Care Services, Institute of Medicine of the National Academies (2004). Insuring America's Health: Principles and Recommendations..

[26] Smedley BD, Stith AY, Nelson AR, Committee on Understanding and Eliminating Racial and Ethnic Disparities in Health Care (2003). Unequal Treatment: Confronting Racial and Ethnic Disparities in Health Care..

[27] Chin MH, Walters AE, Cook SC, Huang ES (2007). Interventions to reduce racial and ethnic disparities in health care.. Med Care Res Rev.

[28] Marmot M (2007). Achieving health equity: from root causes to fair outcomes.. Lancet.

[29] U.S. Department of Health and Human Services, Agency for Healthcare Research and Quality

[30] Ezzati M, Lopez AD, Rodgers A, Hoorn SV, Murray CJL (2002). Selected major risk factors and global and regional burden of disease.. Lancet.

[31] Ezzati M, Hoorn SV, Rodgers A, Lopez AD, Mathers CD (2003). Estimates of global and regional potential health gains from reducing multiple major risk factors.. Lancet.

[32] Ni MC, Rodgers A, Pan WH, Gu DF, Woodward M (2004). Body mass index and cardiovascular disease in the Asia-Pacific Region: an overview of 33 cohorts involving 310 000 participants.. Int J Epidemiol.

[33] Bogers RP, Bemelmans WJ, Hoogenveen RT, Boshuizen HC, Woodward M (2007). Association of overweight with increased risk of coronary heart disease partly independent of blood pressure and cholesterol levels: a meta-analysis of 21 cohort studies including more than 300 000 persons.. Arch Intern Med.

[34] Wilson PW, Bozeman SR, Burton TM, Hoaglin DC, Ben-Joseph R (2008). Prediction of first events of coronary heart disease and stroke with consideration of adiposity.. Circulation.

[35] Spiegelman D, Hertzmark E, Wand HC (2007). Point and interval estimates of partial population attributable risks in cohort studies: examples and software.. Cancer Causes Control.

[36] Preston SH, Heuveline P, Guillot M (2001). Demography: measuring and modeling population processes..

[37] Coale A, Guo G (1989). Revised regional model life tables at very low levels of mortality.. Popul Index.

[38] Murray CJ, Kulkarni SC, Ezzati M (2006). Understanding the coronary heart disease versus total cardiovascular mortality paradox: a method to enhance the comparability of cardiovascular death statistics in the United States.. Circulation.

[39] Murray CJ, Dias RH, Kulkarni SC, Lozano R, Stevens GA (2008). Improving the comparability of diabetes mortality statistics in the U.S. and Mexico.. Diabetes Care.

[40] Lu TH, Hsu PY, Bjorkenstam C, Anderson RN (2006). Certifying diabetes-related cause-of-death: a comparison of inappropriate certification statements in Sweden, Taiwan and the USA.. Diabetologia.

[41] Murray CJ, Ezzati M, Lopez AD, Rodgers A, Vander HS (2003). Comparative quantification of health risks conceptual framework and methodological issues.. Popul Health Metr.

[42] Efron B, Tibshirani R (1986). Bootstrap Methods for Standard Errors, Confidence Intervals, and Other Measures of Statistical Accuracy.. Stat Sci.

[43] Harper S, Lynch J, Meersman SC, Breen N, Davis WW (2008). An overview of methods for monitoring social disparities in cancer with an example using trends in lung cancer incidence by area-socioeconomic position and race-ethnicity, 1992-2004.. Am J Epidemiol.

[44] Law MR, Wald NJ, Thompson SG (1994). By how much and how quickly does reduction in serum cholesterol concentration lower risk of ischaemic heart disease?. BMJ.

[45] Lin HH, Ezzati M, Murray M (2007). Tobacco smoke, indoor air pollution and tuberculosis: a systematic review and meta-analysis.. PLoS Med.

[46] Kivimaki M, Shipley MJ, Ferrie JE, Singh-Manoux A, Batty GD (2008). Best-practice interventions to reduce socioeconomic inequalities of coronary heart disease mortality in UK: a prospective occupational cohort study.. Lancet.

[47] Khang YH, Lynch JW, Yang S, Harper S, Yun SC (2009). The contribution of material, psychosocial, and behavioral factors in explaining educational and occupational mortality inequalities in a nationally representative sample of South Koreans: relative and absolute perspectives.. Soc Sci Med.

[48] Khang YH, Lynch JW, Jung-Choi K, Cho HJ (2008). Explaining age-specific inequalities in mortality from all causes, cardiovascular disease and ischaemic heart disease among South Korean male public servants: relative and absolute perspectives.. Heart.

[49] Salomon JA, Nordhagen S, Oza S, Murray CJ (2009). Are Americans feeling less healthy? The puzzle of trends in self-rated health.. Am J Epidemiol.

[50] Diaz VA, Mainous AG, Koopman RJ, Carek PJ, Geesey ME (2005). Race and diet in the overweight: association with cardiovascular risk in a nationally representative sample.. Nutrition.

[51] Cabe-Sellers BJ, Bowman S, Stuff JE, Champagne CM, Simpson PM (2007). Assessment of the diet quality of US adults in the Lower Mississippi Delta.. Am J Clin Nutr.

[52] Popkin BM, Siega-Riz AM, Haines PS (1996). A comparison of dietary trends among racial and socioeconomic groups in the United States.. N Engl J Med.

[53] Corrao G, Bagnardi V, Zambon A, La Vecchia C (2004). A meta-analysis of alcohol consumption and the risk of 15 diseases.. Prev Med.

[54] Franzini L, Ribble JC, Keddie AM (2001). Understanding the Hispanic paradox.. Ethn Dis.

[55] Smith DP, Bradshaw BS (2006). Rethinking the Hispanic paradox: death rates and life expectancy for US non-Hispanic White and Hispanic populations.. Am J Public Health.

[56] Michaud CM, McKenna MT, Begg S, Tomijima N, Majmudar M (2006). The burden of disease and injury in the United States 1996.. Popul Health Metr.

[57] WHO Committee on the Social Determinants of Health (2008) Closing the gap in a generation: health equity through action on the social determinants of health. Final Report of the Commission on Social Determinants of Health.. http://www.who.int/social_determinants/thecommission/finalreport/en/index.html.

[58] House of Commons Health Committee (2009) Health inequalities: third report of session 2008-2009, Volume 1. London: The Stationery Office Limited.. http://www.publications.parliament.uk/pa/cm200809/cmselect/cmhealth/286/286.pdf.

[59] Davis AM, Vinci LM, Okwuosa TM, Chase AR, Huang ES (2007). Cardiovascular health disparities: a systematic review of health care interventions.. Med Care Res Rev.

[60] Peek ME, Cargill A, Huang ES (2007). Diabetes health disparities: a systematic review of health care interventions.. Med Care Res Rev.

[61] Knowler WC, Fowler SE, Hamman RF, Christophi CA, Hoffman HJ (2009). 10-year follow-up of diabetes incidence and weight loss in the Diabetes Prevention Program Outcomes Study.. Lancet.

[62] Elliott P, Marmot M, Dyer A, Joossens J, Kesteloot H (1989). The INTERSALT study: main results, conclusions and some implications.. Clin Exp Hypertens A.

[63] Law MR, Frost CD, Wald NJ (1991). By how much does dietary salt reduction lower blood pressure? I–Analysis of observational data among populations.. BMJ.

[64] Asaria P, Chisholm D, Mathers C, Ezzati M, Beaglehole R (2007). Chronic disease prevention: health effects and financial costs of strategies to reduce salt intake and control tobacco use.. Lancet.

[65] Murray CJ, Lauer JA, Hutubessy RC, Niessen L, Tomijima N (2003). Effectiveness and costs of interventions to lower systolic blood pressure and cholesterol: a global and regional analysis on reduction of cardiovascular-disease risk.. Lancet.

[66] Thomas S, Fayter D, Misso K, Ogilvie D, Petticrew M (2008). Population tobacco control interventions and their effects on social inequalities in smoking: systematic review.. Tob Control.

[67] Chobanian AV, Bakris GL, Black HR, Cushman WC, Green LA (2003). The Seventh Report of the Joint National Committee on Prevention, Detection, Evaluation, and Treatment of High Blood Pressure: the JNC 7 report.. JAMA.

[68] (2003). Report of the expert committee on the diagnosis and classification of diabetes mellitus.. Diabetes Care.

