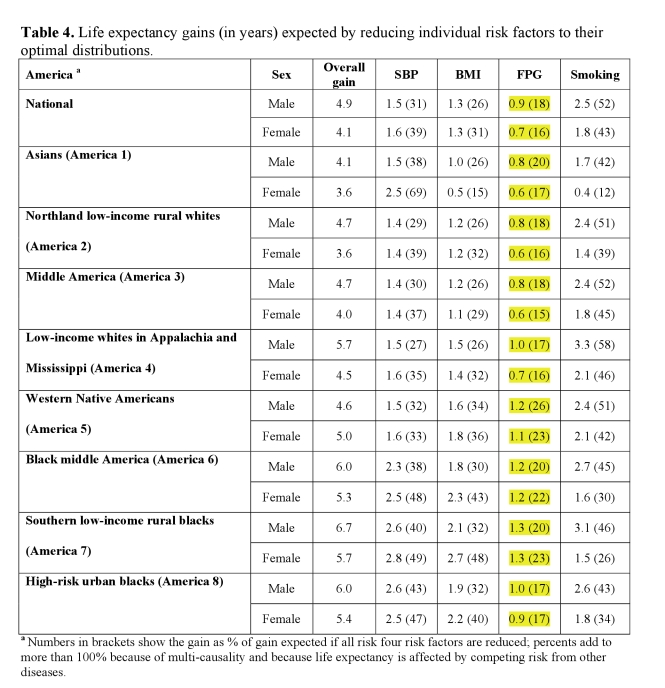# Correction: The Promise of Prevention: The Effects of Four Preventable Risk Factors on National Life Expectancy and Life Expectancy Disparities by Race and County in the United States

**DOI:** 10.1371/annotation/a9616323-4cad-4b65-b792-e0975a536a52

**Published:** 2011-02-01

**Authors:** Goodarz Danaei, Eric B. Rimm, Shefali Oza, Sandeep C. Kulkarni, Christopher J. L. Murray, Majid Ezzati

A coding error led to incorrect estimates for fasting plasma glucose in the sixth column of Table 4. These incorrect numbers are not reported anywhere else in the text, and the results and conclusions of the paper that are based on joint effects of all four risks remain the same. 

Please view the correct Table 4 here: 

**Figure:**
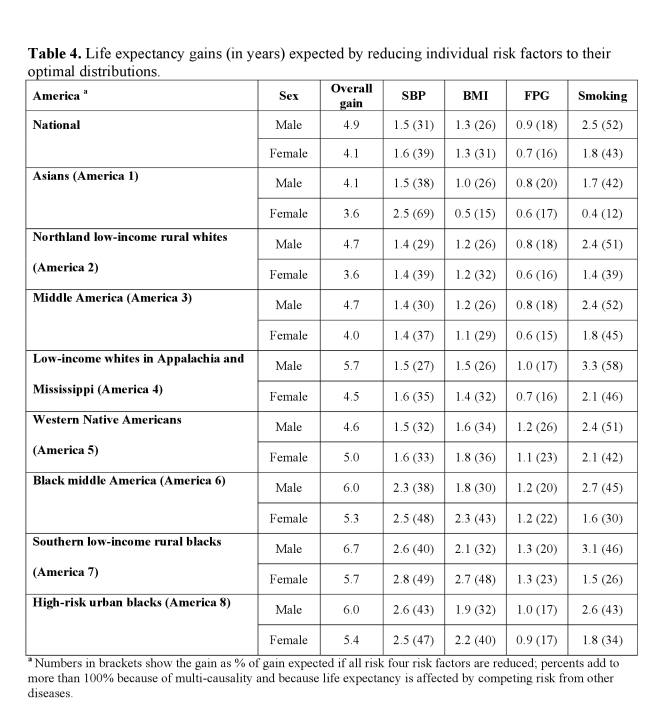


A version of Table 4 with the corrected values highlighted can be found here: 

**Figure:**