# Supplementary material for: The Promise of Prevention: The Effects of Four Preventable Risk Factors on National Life Expectancy and Life Expectancy Disparities by Race and County in the United States
Source: PLoS Med. 2010 Mar 23;7(3):e1000248. doi: 10.1371/journal.pmed.1000248 (PMC2843596; doi:10.1371/journal.pmed.1000248)
Supplement: Table S1 — Evidence on effect size modification by race. (0.07 MB DOC) [file pmed.1000248.s001.doc]

**Table S1**: Evidence on effect size modification by race. ^†^

| **Study** | **Author and year** | **Study design** | **Risk factor or intervention** | **Outcome** | **Subgroups** | **Finding** |
| --- | --- | --- | --- | --- | --- | --- |
| **Observational studies** |  |  |  |  |  |  |
| National Health and Nutrition Examination I Epidemiological Follow-up Study (NHEFS) and NHANES II Mortality Study | Liao 1999 [1] | Population health examination survey data linked to national death index | SBP, smoking and diabetes | Coronary heart disease (CHD) death | Blacks vs. whites | There were no significant differences in the coefficient of the Cox model for the selected risk factors except for smoking in men. |
| Asia-Pacific Cohort Studies Collaboration (APCSC) | APCSC 2004 [2] | Individual-level meta-analysis of 33 prospective cohort studies | BMI | CHD; stroke | Asian vs. Australia and New Zealand (ANZ) cohorts | Relative risks (RRs) in Asian cohorts were not significantly different from Australia-New Zealand cohorts. |
| APCSC | Lawes 2004 [3] | Individual-level meta-analysis of 17 prospective cohort studies | FPG | CHD; stroke | Asian vs. ANZ cohorts | RRs were similar between Asian and ANZ cohorts. |
| INTERHEART | Yusuf 2004 [4] | Multi-center case-control study | smoking, self-reported hypertension and diabetes, waist-hip ratio | Myocardial infarction | Geographical region | RRs of smoking were not significantly different across all regions but those of waist-to-hip ratio and self-reported hypertension and diabetes were. |
| Atherosclerosis Risk In Communities (ARIC) and Cardiovascular Health Study (CHS) | Howard 1997 [5] | Prospective cohort study | Smoking, SBP, diabetes, BMI | Intima Media Thickness | Blacks vs. whites | RRs in blacks were not significantly different from whites for any of the four risk factors |
| Atherosclerosis Risk In Communities (ARIC) | Hozawa 2007 [6] | Prospective cohort study | Smoking, SBP, diabetes, BMI | Myocardial infarction or CHD death | Blacks vs. whites | RRs for having borderline or optimal risk factor status in blacks were not significantly different from whites |
| Charlesto Heart Study | Keil 1993 [7] | Prospective cohort study | SBP, BMI, smoking | CHD death | Blacks vs. whites | Mortality rate ratios were not significantly different between blacks and whites, except for smoking, whose highest rate ratio was in black men and the lowest in black women. |
| ARIC, CHS, Physicians’ Health Study (PHS), Honolulu Heart Program, Puerto Rico Heart Health Program, Strong Heart Study | D’Agostino 2001[8] | Multiple prospective cohort studies | SBP, smoking, diabetes | MI, CHD death | Blacks, white, Japanese Americans, Native Americans and Hispanics | After recalibrating the model for differences in prevalence and underlying CHD rate, the Framingham risk score performed well in all non-white racial/ethnic groups indicating that the proportional effects (RRs) do not depend on race. |
| **Intervention studies** |  |  |  |  |  |  |
| Anti-hypertensive and Lipid Lowering Treatment to Prevent Heart Attack (ALLHAT) | Wright 2005 [9] | Randomized clinical trial comparing different anti-hypertensive drugs | Anti-hypertensive drugs | Fatal CHD, non-fatal MI | Blacks vs non-blacks | RRs for all comparisons of pairs of anti-hypertensive drugs were not significantly different in blacks compared to non-blacks. |
| Action to Control Cardiovascular Risk in  Diabetes (ACCORD) | ACCORD 2008 [10] | Randomized clinical trial comparing intensive glucose lowering to conventional diabetes treatment | Glucose lowering drugs or insulin | Non-fatal MI, non-fatal stroke or CVD death | Blacks vs. whites | There were non-significant differences in the RRs for CVD event or death with blacks showing no reduction in risk. The RRs for death from any cause in blacks were equal to that of whites. |

^†^ These studies were identified using a Pubmed search with keywords related to the corresponding risk factor and race/ancestry separately for prospective studies and for randomized clinical trials.

References

1. Liao Y, McGee DL, Cooper RS (1999) Prediction of coronary heart disease mortality in blacks and whites: pooled data from two national cohorts. Am J Cardiol 84: 31-36.

2. Ni MC, Rodgers A, Pan WH, Gu DF, Woodward M (2004) Body mass index and cardiovascular disease in the Asia-Pacific Region: an overview of 33 cohorts involving 310 000 participants. Int J Epidemiol 33: 751-758.

3. Lawes CM, Bennett DA, Parag V, Woodward M, Whitlock G, et al. (2003) Blood pressure indices and cardiovascular disease in the Asia Pacific region: a pooled analysis. Hypertension 42: 69-75.

4. Yusuf S, Hawken S, Ounpuu S, Dans T, Avezum A, et al. (2004) Effect of potentially modifiable risk factors associated with myocardial infarction in 52 countries (the INTERHEART study): case-control study. Lancet 364: 937-952.

5. Howard G, Manolio TA, Burke GL, Wolfson SK, O'Leary DH (1997) Does the association of risk factors and atherosclerosis change with age? An analysis of the combined ARIC and CHS cohorts. The Atherosclerosis Risk in Communities (ARIC) and Cardiovascular Health Study (CHS) investigators. Stroke 28: 1693-1701.

6. Hozawa A, Folsom AR, Sharrett AR, Chambless LE (2007) Absolute and attributable risks of cardiovascular disease incidence in relation to optimal and borderline risk factors: comparison of African American with white subjects--Atherosclerosis Risk in Communities Study. ArchInternMed 167: 573-579.

7. Keil JE, Sutherland SE, Knapp RG, Lackland DT, Gazes PC, et al. (1993) Mortality rates and risk factors for coronary disease in black as compared with white men and women. N Eng J Med 329: 73-78.

8. D'Agostino RB, Sr., Grundy S, Sullivan LM, Wilson P (2001) Validation of the Framingham coronary heart disease prediction scores: results of a multiple ethnic groups investigation. JAMA 286: 180-187.

9. Wright JT, Jr., Dunn JK, Cutler JA, Davis BR, Cushman WC, et al. (2005) Outcomes in hypertensive black and nonblack patients treated with chlorthalidone, amlodipine, and lisinopril. JAMA 293: 1595-1608.

10. Gerstein HC, Miller ME, Byington RP, Goff DC, Jr., Bigger JT, et al. (2008) Effects of intensive glucose lowering in type 2 diabetes. N Engl J Med 358: 2545-2559.
