# Supplementary material for: The Promise of Prevention: The Effects of Four Preventable Risk Factors on National Life Expectancy and Life Expectancy Disparities by Race and County in the United States
Source: PLoS Med. 2010 Mar 23;7(3):e1000248. doi: 10.1371/journal.pmed.1000248 (PMC2843596; doi:10.1371/journal.pmed.1000248)
Supplement: Table S2 — Studies on the proportion of BMI-CVD excess risk mediated through blood pressure and blood glucose/diabetes. (0.06 MB DOC) [file pmed.1000248.s002.doc]

**Table S2**: Studies on the proportion of BMI-CVD excess risk mediated through blood pressure and blood glucose/diabetes. ^†^

| **Study** | **Author and year** | **Study design** | **Outcome** | **Mediators considered** | **Results after adjustment for mediators** |
| --- | --- | --- | --- | --- | --- |
| Prospective studies of healthy subjects (mostly white) | Bogers 2007 [1] | Meta-analysis of 15 prospective cohort studies | Coronary heart disease (CHD) | SBP; total cholesterol (TC) | Excess risk decreased by 46% per unit BMI. |
| Framingham Offspring cohort | Wilson 2008 [2] | Prospective cohort | CHD and cerebrovascular accident (CVA) | SBP; total/HDL cholesterol ratio; self-reported diabetes | Excess risk per unit BMI decreased by 25% for both CHD and CVA with adjustment for SBP. The reduction in excess risk of CHD was 64% after adjustment for SBP, total/HDL ratio and diabetes. |
| Follow-up of 2 cross-sectional population surveys in Finland | Jousilahti 1996 [3] | Prospective cohort | CHD mortality | SBP | Excess risk per unit BMI decreased by 50% in men and 40% in women. |
| Follow-up of 3 cross-sectional population surveys in Finland | Hu 2004 [4] | Prospective cohort | CVD | SBP; TC; diabetes | Excess risk of overweight-obesity decreased by 44% to 65% in different categories of BMI by gender |
| Korean cohort of men and women | Jee 2006 [5] | Prospective cohort | CVD | SBP; TC; FPG | Excess risk decreased by 50% to 100% in different categories of BMI. The only group that still had significantly increased risk was obese men with BMI > 32 |
| Follow-up of 6 cross-sectional population surveys in Finland | Hu 2007 [6] | Prospective cohort | Ischemic stroke | SBP; total cholesterol; history of DM | Excess risk decreased by 49% in the obese and 45% in the overweight category. |
| Women’s Health Study | Kurth 2005 [7] | Prospective cohort | Ischemic stroke | History of hypertension, DM and elevated total cholesterol | Excess risks decreased by 55% in the very obese, and 70% in the obese (BMI 30-35) and 18% in the overweight category (BMI of 27-30) (all compared to BMI<22) |

**^†^** We searched Pubmed for prospective studies of BMI and coronary heart disease or stroke, that also had “blood pressure”, “blood glucose” or “diabetes” in their Medical Subject Heading (MeSH) terms.

**^‡^** Only one study examined mediation by blood glucose as a continuous variable [5]. The other studies used diagnosed or self-reported diabetes and are therefore likely to underestimate the role of blood glucose as a mediator of risk for BMI.

References

1. Bogers RP, Bemelmans WJ, Hoogenveen RT, Boshuizen HC, Woodward M, et al. (2007) Association of overweight with increased risk of coronary heart disease partly independent of blood pressure and cholesterol levels: a meta-analysis of 21 cohort studies including more than 300 000 persons. ArchInternMed 167: 1720-1728.

2. Wilson PW, Bozeman SR, Burton TM, Hoaglin DC, Ben-Joseph R, et al. (2008) Prediction of first events of coronary heart disease and stroke with consideration of adiposity. Circulation 118: 124-130.

3. Jousilahti P, Tuomilehto J, Vartiainen E, Pekkanen J, Puska P (1996) Body weight, cardiovascular risk factors, and coronary mortality. 15-year follow-up of middle-aged men and women in eastern Finland. Circulation 93: 1372-1379.

4. Hu G, Tuomilehto J, Silventoinen K, Barengo N, Jousilahti P (2004) Joint effects of physical activity, body mass index, waist circumference and waist-to-hip ratio with the risk of cardiovascular disease among middle-aged Finnish men and women. Eur Heart J 25: 2212-2219.

5. Jee SH, Sull JW, Park J, Lee SY, Ohrr H, et al. (2006) Body-mass index and mortality in Korean men and women. NEnglJMed 355: 779-787.

6. Hu G, Tuomilehto J, Silventoinen K, Sarti C, Mannisto S, et al. (2007) Body mass index, waist circumference, and waist-hip ratio on the risk of total and type-specific stroke. ArchIntern Med 167: 1420-1427.

7. Kurth T, Gaziano JM, Rexrode KM, Kase CS, Cook NR, et al. (2005) Prospective study of body mass index and risk of stroke in apparently healthy women. Circulation 19;111: 1992-1998.
