# Supplementary material for: The Promise of Prevention: The Effects of Four Preventable Risk Factors on National Life Expectancy and Life Expectancy Disparities by Race and County in the United States
Source: PLoS Med. 2010 Mar 23;7(3):e1000248. doi: 10.1371/journal.pmed.1000248 (PMC2843596; doi:10.1371/journal.pmed.1000248)
Supplement: Text S1 — Calculating PAF for multiple risks, including mediated effects. (0.04 MB DOC) [file pmed.1000248.s003.doc]

**Text S1:** Calculating PAF for multiple risks, including mediated effects

When exposure to a risk factor is uncorrelated with those of other risk factors that affect the same disease, the PAF for individual diseases can be calculated using the simple relationship below. The PAF for multiple uncorrelated risks can also be calculated using a simple relationship from the PAFs of individual risks.

*RR*(*x*): relative risk at exposure level *x*, for the causal effect of risk factor on disease-specific mortality

*P*(*x*): actual distribution of risk factor exposure in the population

*P*′(*x*): alternative distribution of exposure relative to which effects are estimated, e.g. an optimal exposure distribution

*m*: maximum exposure level.

As described in Methods, to incorporate risk factor correlation we computed the PAF for multiple risks by summing their combined (joint) RR for individual records, weighted by their sampling probability.

Few large prospective studies were powered to examine the RRs for the joint effects of multiple CVD risk factors in our analysis. These studies do not provide unequivocal support on whether multiple risks interact in a multiplicative vs. sub-multiplicative (e.g. additive excess risk) manner [1-6]. To calculate to joint RR for the effects of these risks we used an additive excess risk scale, because it gives a more conservative estimate of the combined effects than a multiplicative model. We also incorporated the partial mediation of the effects of BMI on CVD through SBP and FPG.

The above issues can be represented using the following relationship:

$$RR_{SPGB}=1+(e^{\beta_{B}(L_{B}-T_{B})}-1)\cdot\left( 1-\alpha\right)+\sum_{i=SPG} (e^{\beta_{i}(L_{i}-T_{i})}-1)$$

Where S, P, G and B represent smoking, SBP, FPG and BMI respectively; α is the proportion of excess risk of BMI mediated through SBP and FPG and is obtained from the pooled analysis of prospective cohort studies as discussed in the Methods (α would be zero if none of the BMI effects were mediated through SBP and FPG, e.g. for cancers; it is between 0 and 1 if a part of BMI effects are mediated through these factors, e.g. for ischemic heart disease, IHD); L is the current exposure level for each record; T is the alternative exposure level; and β is the log RR per unit exposure (or category of exposure for smoking).

Using multiplicative scale for joint RR estimation had little effect on the results. Specifically, multiplicative RRs led to 0.1-0.6 years larger life expectancy gains across the Eight Americas than the additive RRs.

Reference List

1. Whitlock G, Lewington S, Sherliker P, Clarke R, Emberson J, et al. (2009) Body-mass index and cause-specific mortality in 900 000 adults: collaborative analyses of 57 prospective studies. Lancet 373: 1083-1096.

2. Stratton IM, Cull CA, Adler AI, Matthews DR, Neil HA, et al. (2006) Additive effects of glycaemia and blood pressure exposure on risk of complications in type 2 diabetes: a prospective observational study (UKPDS 75). Diabetologia 49: 1761-1769.

3. Neaton JD, Wentworth D (1992) Serum cholesterol, blood pressure, cigarette smoking, and death from coronary heart disease. Overall findings and differences by age for 316,099 white men. Multiple Risk Factor Intervention Trial Research Group. Arch InternMed 152: 56-64.

4. Pednekar MS, Gupta PC, Hebert JR, Hakama M (2008) Joint effects of tobacco use and body mass on all-cause mortality in Mumbai, India: results from a population-based cohort study. Am J Epidemiol 167: 330-340.

5. Al Delaimy WK, Manson JE, Solomon CG, Kawachi I, Stampfer MJ, et al. (2002) Smoking and risk of coronary heart disease among women with type 2 diabetes mellitus. Arch InternMed 162: 273-279.

6. Hu G, Jousilahti P, Tuomilehto J (2007) Joint effects of history of hypertension at baseline and type 2 diabetes at baseline and during follow-up on the risk of coronary heart disease. EurHeart J 28: 3059-3066.
